# Numerical and Experimental Study on Carbon Segregation in Square Billet Continuous Casting with M-EMS

**DOI:** 10.3390/ma16165531

**Published:** 2023-08-09

**Authors:** Pengchao Li, Guifang Zhang, Peng Yan, Peipei Zhang, Nan Tian, Zhenhua Feng

**Affiliations:** 1 Faculty of Metallurgical and Energy Engineering, Kunming University of Science and Technology, Kunming 650093, China; 13696392999@163.com (P.L.); zpei861@163.com (P.Z.); tian1852558@163.com (N.T.); fengzhenhua666@126.com (Z.F.); 2 Linyi Iron and Steel Investment Group Special Steel Co., Ltd., Linyi 276000, China; 3 Key Laboratory of Clean Metallurgy for Complex Iron Resources in Colleges and Universities of Yunnan Province, Kunming University of Science and Technology, Kunming 650093, China

**Keywords:** M-EMS, continuous casting billet, multi-physical field model, carbon segregation

## Abstract

Electromagnetic stirring (M-EMS) has been extensively applied in continuous casting production to reduce the quality defects of casting billets. To investigate the effect of continuous casting electromagnetic stirring on billet segregation, a 3D multi-physics coupling model was established to simulate the internal heat, momentum, and solute transfer behavior, to identify the effect of M-EMS on the carbon segregation of a continuous casting square billet of 200 mm × 200 mm. The results show that M-EMS can move the high-temperature zone upward, which is favorable for the rapid solidification of the billet, and can promote the rotational flow of the molten steel in the horizontal direction. When the electromagnetic stirring current is varied in the range of 0–500 A, the degree of carbon segregation first decreases and then increases, with the best control of segregation at 300 A. In the frequency range of 3–5 Hz, the degree of carbon segregation degree increases with frequency. Meanwhile, the simulation and experimental results show that 3 Hz + 300 A is the best electromagnetic stirring parameter for improving the carbon segregation of casting billets with a size of 200 mm × 200 mm. So, a reasonable choice of the M-EMS parameters is crucial for the quality of the billet.

## 1. Introduction

Continuous casting is an essential link in the production of high-quality steel and special steel. The segregation behavior of continuous casting billets has an essential impact on the internal quality and performance uniformity of subsequent steel products [1,2]. The continuous casting process of the billet is dominated by highly complex and interrelated phenomena, including molten steel flow, heat transfer, solute transport, solidification nucleation, and crystal growth and transport [3,4]. For the typical segregation behavior in the continuous casting process, the reported causes include the flow, solute transfer, and relative motion of the solid and liquid phases. Electromagnetic stirring (EMS) is a process that uses electromagnetic force to stir processed molten metal. It generates an alternating magnetic field through a copper coil and an electromagnetic force through the interaction of an induced current and magnetic field in a liquid metal [5]. It can be used to control the flow, heat transfer, and solidification of molten steel by electromagnetic forces, thus enabling optimization of the casting process [6]. Electromagnetic stirring in mold (M-EMS) technology is a vital application form of EMS, which is an effective means to optimize the flow of molten steel in the continuous casting process. It is widely used in the continuous casting production of high-quality and unique steel. The slab’s solidification structure, center segregation, and inclusion distribution can be improved by controlling the flow and heat transfer of molten steel. However, M-EMS also has some adverse consequences, such as negative segregation [7]. This segregation, also known as a white band, will affect the hardenability, surface hardness, and mechanical properties of steel, and it is often challenging to eliminate in the subsequent process of continuous casting [8,9]. There is no unified explanation for the formation mechanism of negative segregation bands [10], and the widely accepted theory is mainly the solute washing mechanism. It is believed that the electromagnetic force makes the molten steel flow, flushing the solidification front and removing the solute-enriched molten steel [11,12]. In this case, a negative segregation band is formed. Kor et al. [13] reported that when the billet enters and leaves the electromagnetic stirring zone, the billet’s sudden slow and fast solidification rate is the main reason for negative segregation bands. Ayata et al. [14] found that the subcutaneous negative segregation of the continuous casting billet gradually deteriorated with the increase in M-EMS intensity. It is considered that the electromagnetic stirring drives the flow of molten steel and the impact on the nozzle. These phenomena bring the high-concentration solute discharged from the solidification front to the central liquid phase area, causing the formation of subcutaneous negative segregation. Wu et al. [15] reported that the carbon segregation index reduced from 1.412 to 1.201 with electromagnetic torque of M-EMS from 230 to 400 cN·cm. Tao et al. [10] found that a suitable current of EMS could reduce the carbon segregation in industrial tests, and the segregation became severe with the progressive increase in current. The segregation behavior in the continuous casting process involves multiple processes, such as molten steel flow, solute migration, heat transfer, and solidification [16]. For molten steel’s opacity and high-temperature characteristics, the liquid phase flow state and solute transport behavior of molten steel would not be directly understood by traditional casting industrial tests. Therefore, numerical simulation methods have been adopted by many researchers to carry out relevant studies [17]. Jiang et al. [9] reported that M-EMS with a higher stirring current would not continue to increase the equiaxed crystal ratio of the continuous casting billet, but more serious negative segregation appears. The effects of equiaxed grain settlement and liquid hot melt flow lead to negative segregation bands and positive segregation near the center of the billet. Okazawa et al. [18] adopted a numerical simulation method to quantitatively describe the effect of EMS on the molten steel flow structure by using the homogeneity index and found that the different installation positions and logarithms of agitators would significantly impact the flow structure in the mold.

Sun et al. [19] established a segmented three-dimensional electromagnetic–thermal coupling solute transport model to better understand the macroscopic segregation formation in the billet during continuous casting. The effect of M-EMS operating parameters on the segregation was studied by conducting industrial tests. It was found that the simulated W-shaped segregation distribution along the casting thickness was in good agreement with the measured distribution. Zhang et al. [20] used a multi-physical field numerical model to study the macroscopic transfer behavior in the billet. Then, it was found that with the increase in M-EMS current density, the high-temperature region would move upward, and the negative segregation under the skin would finally become more severe due to the intense flushing of the initial solidified shell caused by the flow. Yu et al. [21] numerically simulated the magnetic field, temperature field, and inclusion trajectory of M-EMS under different parameters for round billet continuous casting. Based on the numerical simulation results, industrial experiments were carried out to investigate the solidification structure obtained under different M-EMS parameters and the macro-segregation behavior of high-carbon steel during continuous casting. Fang et al. [22] established a multi-physical field numerical model to investigate the flow, temperature field, and solute concentration field in bloom (380 mm × 280 mm) casting under the action of M-EMS. It was found that M-EMS made the distribution of temperature, solute, and solidified shell more uniform. However, M-EMS has not been found to improve the central segregation of this bloom.

In order to explore the causes and improvement measures of segregation in continuous casting, many researchers have developed multi-physical field models to reveal the macroscopic transport phenomena of the continuous casting electromagnetic stirring process. However, due to the differences in steel grades and equipment in the continuous casting production processes, a unified conclusion has not yet been formed to fully understand the heat transfer, flow structure, and solute distribution of molten steel under the action of M-EMS. Therefore, in this paper, the electromagnetic–thermal-flow multi-physical field coupling model is established to simulate the temperature field, flow field, and distribution characteristics of carbon in the molten steel of a 200 mm × 200 mm billet, and to explore the influence of M-EMS on the segregation behavior of the billet under different parameters, to provide a technical reference for continuous casting production.

## 2. Model Description

### 2.1. Assumptions

The flow and heat transfer in the numerical model of EMS correlates with the magnetic field. Thus, the Navier–Stokes, Maxwell, heat transfer, and solute transport equations should be coupled solutions to the quantitative analysis of the transport phenomena in the mold. To simplify the complexity and enhance computational efficiency, the assumptions are as follows:

1. The steel in the calculation domain is considered as incompressible Newtonian viscous fluid;
2. The computing domain is considered vertical and the influence of mold oscillation and taper is ignored;
3. The electromagnetic field in the effective zone of M-EMS does not affect the molten steel flow;
4. While solving the solute diffusion, the interaction of solute elements can be ignored;
5. The heat transfer phenomenon between the molten steel and the top slag of the meniscus is ignored.

### 2.2. Governing Equations

(1) Fluid Flow Model

The continuity and momentum equations for the melt flow pattern under turbulent conditions can be described as follows:

(1) ∇·U=0

(2) $$\frac{\partial }{\partial t}(\rho U)+\nabla \cdot (\rho UU)=\nabla \cdot ((\mu_{l}+\mu_{t})\nabla \cdot U)-\nabla P+S_{p}+F_{m}$$

where *U* denotes flow velocity (m/s), *μ_l_* denotes laminar viscosity (kg/m^−1^·s^−1^), *μ_t_* denotes turbulent viscosity (kg/m^−1^·s^−1^), *ρ* denotes density (kg/m^3^), *P* denotes static pressure (Pa), *S_p_* denotes momentum sink, and *F_m_* denotes electromagnetic force (N/m^3^). The standard κ−ε model was used to describe the turbulent flow of molten steel as follows:

(3) $$\frac{\partial }{\partial_{t}}\left(\rho \kappa \right)+\nabla \cdot \left(\rho U\kappa \right)=\nabla \cdot \left[\left(\mu_{l}+\frac{U_{t}}{\sigma_{k}}\right)\nabla \kappa \right]+G_{k}+G_{b}-\rho \varepsilon +S_{k}$$

(4) $$\frac{\partial \left(\rho \varepsilon \right)}{\partial_{t}}+\nabla \cdot \left(\rho U\varepsilon \right)=\nabla \cdot \left[\left(\mu_{l}+\frac{U_{t}}{\sigma_{\varepsilon }}\right)\nabla \varepsilon \right]+C_{1\varepsilon }\frac{\varepsilon }{\kappa }\left(G_{\kappa }+C_{3\varepsilon }G_{b}\right)-C_{2\varepsilon }\rho \frac{\varepsilon^{2}}{\kappa }+S_{\varepsilon }$$

*G_k_* denotes the turbulence kinetic energy referring to mean velocity gradients, *G_b_* denotes the turbulence kinetic energy referring to buoyancy, and *μt* is the turbulent viscosity. $C_{1\varepsilon }$, $C_{2\varepsilon }$, $C_{3\varepsilon }$, and $C_{\mu }$ are constant and $\sigma_{k}$ and $\sigma_{\varepsilon }$ are the turbulent Prandtl numbers for κ and ε, respectively. $C_{1\varepsilon }$, $C_{2\varepsilon }$, $C_{\mu }$, $\sigma_{k}$, and $\sigma_{\varepsilon }$ are recommended to be 1.44, 1.92, 0.09, 1.0, and 1.3, respectively [23]. When considering the effects of the porous material, $S_{k}$ and $S_{\varepsilon }$ denote the source terms.

(2) Heat Transfer Model

The following equation can describe the energy conservation during the continuous casting process:

(5) $$\frac{\partial }{\partial t}(\rho H)+\nabla \cdot (\rho UH)=\nabla \cdot (k_{eff}\nabla T)$$

where *k_eff_* denotes the effective thermal conductivity (W·m^−1^·K^−1^), and *T* and *H* denote the temperature (K) and the total enthalpy, respectively.

(3) Solute Transport Model

The solute distribution is affected by Fickian diffusion, convection, and phase transport. The solute conservation equation is used to describe the solute transport phenomenon as follows:

(6) $$\begin{array}{cc} \frac{\partial (\rho C_{i})}{\partial t}+\nabla \cdot (\rho UC_{i}) & =\nabla \cdot (\rho f_{s}D_{s,i}\nabla C_{s,i})+\nabla \cdot (f_{l}(\rho D_{l,i}+\frac{\mu_{t}}{Sc_{t}})\nabla C_{l,i})\\ & -\nabla \cdot (\rho f_{s}(U-U_{s})(C_{l,i}-C_{s,i})) \end{array}$$

where $C_{l,C}$ and $D_{s,C}$ (m^2^/s) denote the solute diffusion coefficients of solute elements in liquid and solid phases [24].

(4) Electromagnetic Field Model

Maxwell’s equations can describe the electromagnetic field during continuous casting with M-EMS:

(7) $$\nabla \times H=J+\frac{\partial D}{\partial t}=J_{s}+J_{e}+\frac{\partial D}{\partial t}$$

(8) $$\nabla \times E=-\frac{\partial B}{\partial t}$$

(9) ∇·B=0

(10) ∇·D=ρ

where *H* (A⋅m^−1^) denotes magnetic field strength, *D* (C·m^−2^) denotes electric flux density, J (A⋅m^−2^) denotes electric current density, *E* (V·m^−1^) denotes electric field strength, *B* (T) denotes magnetic flux density, and ρ (C·m^−3^) denotes electric charge density.

Then, the time-averaged electromagnetic force can be calculated by:

(11) $$F_{m}=0.5Re(J\times B)$$

where *F_m_* is the time-averaged electromagnetic force and *Re* represents the genuine part of a complex number.

### 2.3. Model Parameters and Boundary Conditions

**Figure 1:**
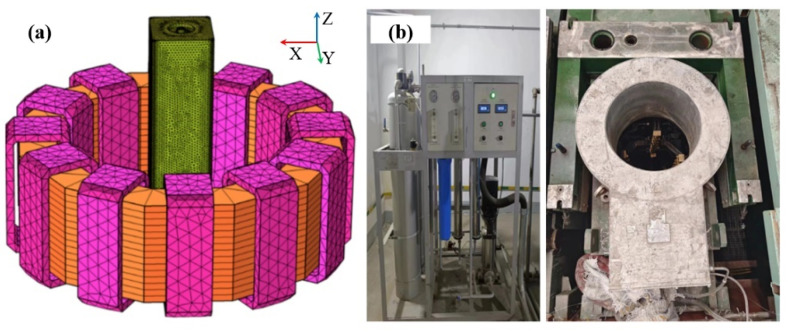
(**a**) Schematic diagram of model mesh division; (**b**) equipment photos.

The parameters of the continuous casting and the physical properties of molten steel are given in Table 1. The finite element model consists of an electromagnetic agitator, a toroidal core, and 12 coil packages with a 30 degree symmetry distribution. In order to ensure the accuracy of the calculations, the area near the mold walls and the outlet of the submerged water is encrypted with grids. The total number of meshes in the model is about 500,000, and the model mesh partition and device photos are shown in Figure 1.

## 3. Experimental Implementation

**Figure 2:**
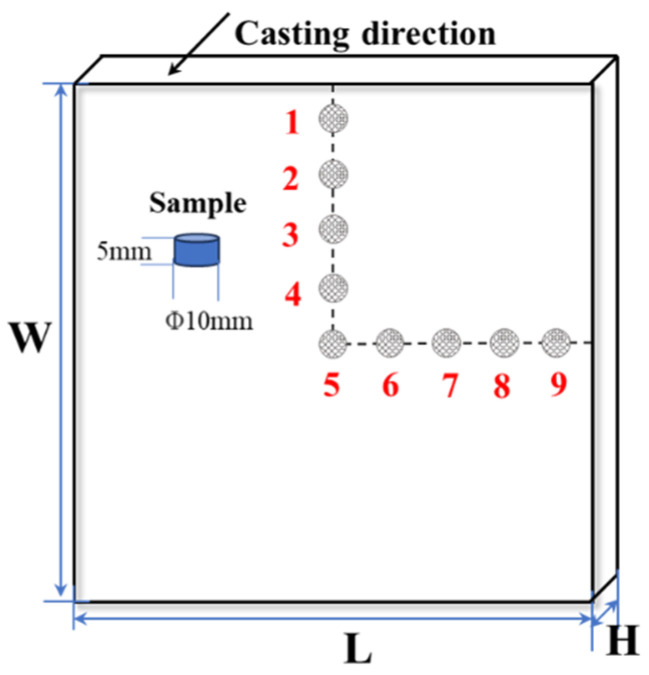
Sampling diagram of the influence of M−EMS on carbon segregation in casting billet.

A schematic of the billet continuous casting and picture of the M-EMS are shown in Figure 1. The M-EMS with a Klem winding was installed at 0.5 m below the meniscus when the 200 mm × 200 mm carbon steel was produced. A φ10 mm drill bit was used to sample the cross−section of the continuous casting billet, and 9 points were taken on each continuous casting section (the length of the billet is L = 200 mm; the width is W = 200 mm; the thickness is H = 10 mm). Point 5 is the central point, point 2 is W/4, point 3 is W/2, point 4 is W3/4, point 6 is L/4, point 7 is L/2, point 8 is L3/4, and point 1 and point 9 are 10 mm under the skin of the edge of the casting billet, respectively. The sampling diagram is shown in Figure 2, in which the billet sample for analysis was drilled with a tungsten steel drill bit. Carbon content analysis of the billet section in the transverse and longitudinal directions was conducted to evaluate the carbon segregation of the billet. Carbon concentration at the billet subsurface of each point was analyzed with a carbon–sulfur analyzer (EMIA Pro, Horiba Inc., Kyoto, Japan). For this research, 30Cr13 steel was used, and the main chemical components are listed in Table 2.

In order to investigate the variation of segregation of the carbon steel, the segregation index was used to characterize the macroscopic segregation of the billet. The carbon segregation index of the billet is calculated according to Equation (12) [27]:

(12) $$a=\frac{C_{i}}{C_{x}}$$

where a is the element segregation index; $C_{i}$ is the element concentration at the test position *i*; and $C_{x}$ is the average element concentration at all detection locations on the specimen section.

## 4. Results and Discussion

### 4.1. Model Validation

**Figure 3:**
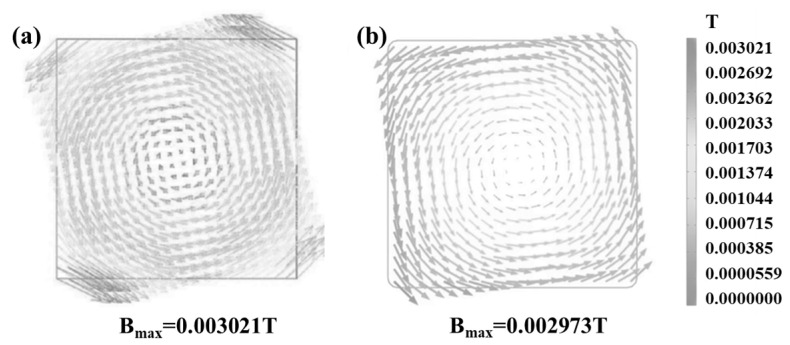
Distribution of magnetic induction intensity inside continuous casting billet under electromagnetic stirring: (**a**) Results of literature reports [28]; (**b**) calculation results of this study.

To verify the reliability of the multi−physical field model describing continuous casting with M−EMS, the internal magnetic distribution results of the billet at the parameters of 240 A current and 4 Hz frequency are presented in Figure 3b. In addition, they are compared with the results reported in the literature under the same conditions [28], as presented in Figure 3a. Then, it can be identified that the minimum flux density predicted by the numerical model in this research occurs in the center of the billet, the magnetic flux density is evenly distributed on the cross−section of the billet, the electromagnetic force rotates along the cross−section, the maximum magnetic flux density is located at the billet corner, and the minimum magnetic flux density is located at the center of the billet. The magnetic field profiles calculated with the model in this paper are consistent with the structure and trends of the magnetic field distribution in the comparative literature. The maximum induced magnetic field intensity calculated by the simulation in this paper is 0.002973 T, while the maximum induced magnetic field intensity in the comparison literature is 0.003021 T. The relative error between the two values for maximum induced magnetic field intensity is only 1.59%, which indicates that the model established in this study can be used for magnetic field prediction. It can be used to assess the influence of EMS on the billet continuous casting process.

### 4.2. Melt Heat Transfer

**Figure 4:**
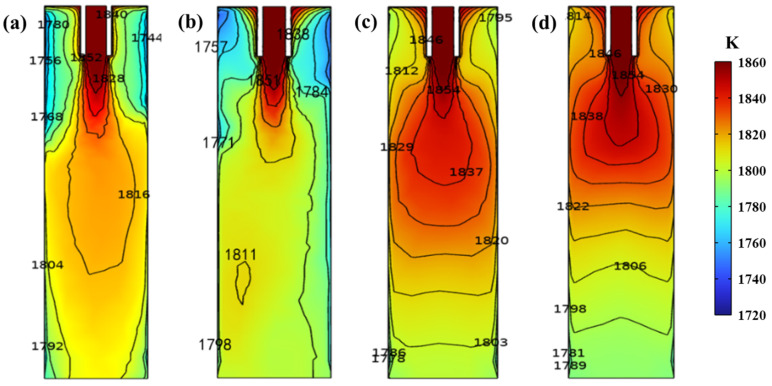
Effect of current on the temperature field of the vertical central section in the continuous casting mold: (**a**) without M−EMS; (**b**) 100 A; (**c**) 300 A; (**d**) 500 A.

The temperature field distribution of the vertical center section of the mold under different M−EMS currents is shown in Figure 4. In the beginning, without the action of M−EMS, the molten steel is directed diagonally into the inner cavity of the mold from the ingoing nozzle, and the molten steel diverges around the lower part. Then, the upward reflux of molten steel is reduced and the temperature is lower, leading to a significant temperature drop in the lower part of the meniscus and around the inlet, where the temperature is lower than in other areas. At the same time, the temperature range of the molten steel below the inlet does not favor the solidification of the casting billet. As the action of M−EMS at the current increases from 100 A to 500 A, the temperature of the molten steel around the inlet increases appreciably. At a current of 100 A, the region of influence of the induced magnetic field is limited, and the stirring effect acts only on the molten steel below the ingoing nozzle. When superimposed with inertia, the upward flow of molten steel in this region is weakened, resulting in heat loss of the molten steel below the meniscus, so the temperature of molten steel below the meniscus is lower than that without M−EMS. As shown in Figure 4, the temperature of the molten steel at a current of 300 A is most uniform in the same horizontal plane near the outlet of the mold, and the superheating of the molten steel can be effectively reduced or even eliminated.

**Figure 5:**
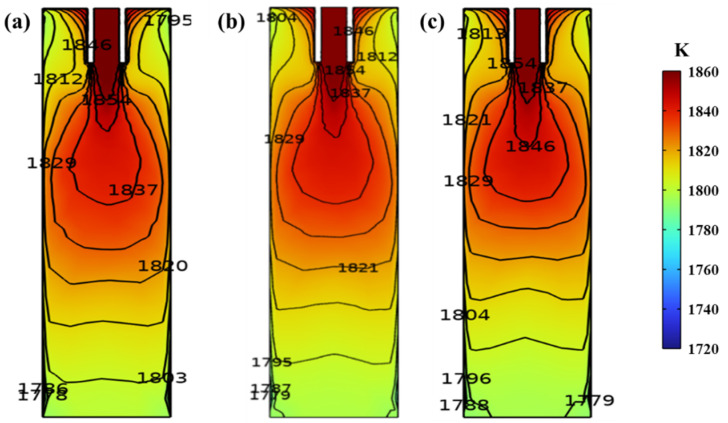
Effect of frequency on the temperature field of the vertical central section in the continuous casting mold: (**a**) 3 Hz; (**b**) 4 Hz; (**c**) 5 Hz.

Figure 5 shows the temperature field distribution of the vertical central section of the mold at different frequencies, where the current is set to 300 A. As the frequency increases from 3 Hz to 5 Hz, the temperature of the molten steel around the inlet of the mold first decreases and then increases, with values of 1837 K, 1829 K, and 1846 K, respectively. Then, a significant upward shift of the high−temperature zone can be seen, which favors a shortening of the solidification time of the casting billet. At the same time, the high−temperature zone of the molten steel is gradually confined to the upper part of the mold, and more of the heat of the molten steel is likely to be carried away by cooling water in the continuous casting mold. By comparing Figure 5a–c, the isothermal region edge of 1854 K is closer to the submerged entry nozzle with increasing frequency from 3 Hz to 5 Hz. Around the meniscus, the temperature of the molten steel increases with the increase in M−EMS frequency, with values of 1795 K, 1804 K, and 1813 K, respectively. At this moment, the maximum temperature difference is 18 K, and the temperature of the molten steel rises significantly with increasing frequency. When the frequency exceeds 3 Hz, the temperature in the center of the casting billet near the mold outlet is the lowest in the same horizontal plane, which may not be favorable for the solidification of the casting billet. Then, the excessive temperature gradient impairs the formation of an equiaxed crystal structure, which is unfavorable for uniform solute distribution [29].

### 4.3. Melt Flow Field

**Figure 6:**
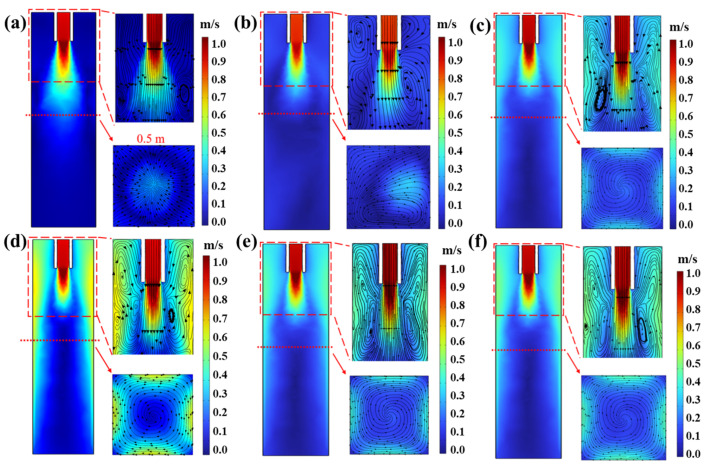
Flow field distribution in the mold under different M−EMS parameters: (**a**) without M−EMS; (**b**) 3 Hz + 100 A; (**c**) 3 Hz + 300 A; (**d**) 3 Hz + 500 A; (**e**) 4 Hz + 300 A; (**f**) 5 Hz + 300 A.

Figure 6 shows the distribution of the flow field in the mold under different current and frequency parameters. When the molten steel enters the inner cavity of the mold from the inlet, the molten steel diffuses without M−EMS, and the velocity gradually decreases, forming a narrow vortex at the lower left and right sides of the mold inlet. The flow field at the horizontal section of the center of the electromagnetic stirring device shows that the velocity distribution of the steel flow in the billet center is divided into three parts: the velocity is highest in the center of the billet, followed by the area near the surface of the billet, and the area with the lowest velocity is located between the center and the surface of the billet. When the M−EMS current is 100 A, the vortices on the left and right sides below the inlet are shifted upward, and a minor vortex is gradually formed below the bending moon surface, which is conducive to complete mixing and an improvement in the quality of the casting billet. At the same time, the horizontal profile of the flow field at the center of the electromagnetic stirring 0.5 m below the Mencius scale indicates that the induced magnetic field has a stirring effect on the molten steel. Due to the limited stirring effect of the electromagnetic force generated by the slight current on the molten steel, the molten steel forms a clockwise vortex on the horizontal surface, and the area with a higher flow speed is still located in the billet. As the current gradually increases to 300–500 A, the vortices in the lower part of the inlet gradually move upward and fuse with the vortices in the lower part of the meniscus, eventually forming giant vortices. According to the distribution characteristics of the magnetic field, the electromagnetic force acting on the molten steel near the mold walls is more significant. At the same time, the flow rate of the molten steel near the walls of the mold also increases and is higher than that of the molten steel at the center of the billet and at the four corners of the mold.

Compared with Figure 6a, Figure 6c,e,f show that a large vortex flow is generated near both sides of the mold inlet with a frequency of 3–5 Hz, and the influence area of the vortex flow can reach near the meniscus. Then, the vortex flow can accelerate the entire flow of molten steel. At the same time, the flow field in the horizontal section of the center of the stirrer 0.5 m below the curved moon indicates that the magnetic field generated by M−EMS has a horizontal rotational stirring effect on the steel. The electromagnetic forces acting on the steel near the mold walls are more significant than at the center of the billet, and the steel flow rate near the mold walls is higher than at the center of the billet and at the corners of the mold. This distribution of electromagnetic force contributes to a higher stirring speed in the area near the billet walls, which is consistent with the results reported in the literature [22], with the different maximum velocities for the different stirring currents. Then, it will facilitate the full mixing flow of the molten steel and promote the formation of equiaxed grain, which is beneficial to the homogenization of temperature and solute components in the molten steel [30].

**Figure 7:**
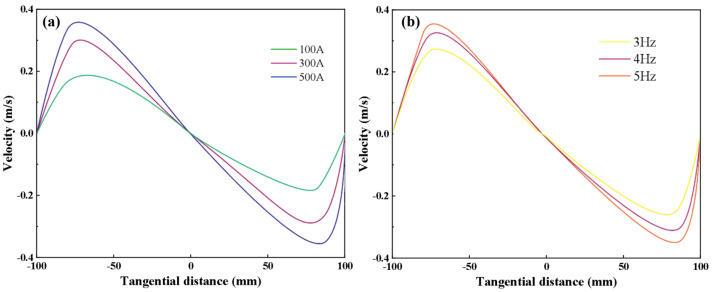
Influence of M−EMS on tangential velocity of molten steel in central cross−section: (**a**) Current at 100, 300, 500 A of 3 Hz; (**b**) frequency at 3, 4, 5 Hz of 300 A.

Figure 7 shows the curve of the tangential velocity of the molten steel in the central cross−section of the electromagnetic agitator at 0.5 m below the meniscus for different current intensities and frequencies. The tangential velocity of molten steel in the flowing area increases with the increase in the distance from the center of the billet, which is beneficial to promoting the rotational flow of the steel in the horizontal plane [31,32]. This is because the electromagnetic force generated by the electromagnetic agitator on molten steel increases with the increase in the distance from the center of the billet. Moreover, the velocity gradually decreases before approaching the casting walls due to the wall effect. Then, the tangential velocity of the molten steel near the walls gradually decreases until it reaches zero. Meanwhile, with the increase in current intensity, the maximum tangential velocity of molten steel is 0.19, 0.31, and 0.38 m/s at 100 A, 300 A, and 500 A, respectively. Because the electromagnetic force near the mold walls area is large and the electromagnetic force away from the mold walls area is small, the flow trend of the molten steel cannot be changed by the simple adjustment of the M−EMS current or frequency parameters; this can affect only the flow speed of the liquid steel. Thus, as the M−EMS frequency is increased from 3 Hz to 5 Hz, the maximum tangential velocities of the molten steel are 0.31, 0.39, and 0.44 m/s, respectively.

### 4.4. Distribution of Solute Carbon

**Figure 8:**
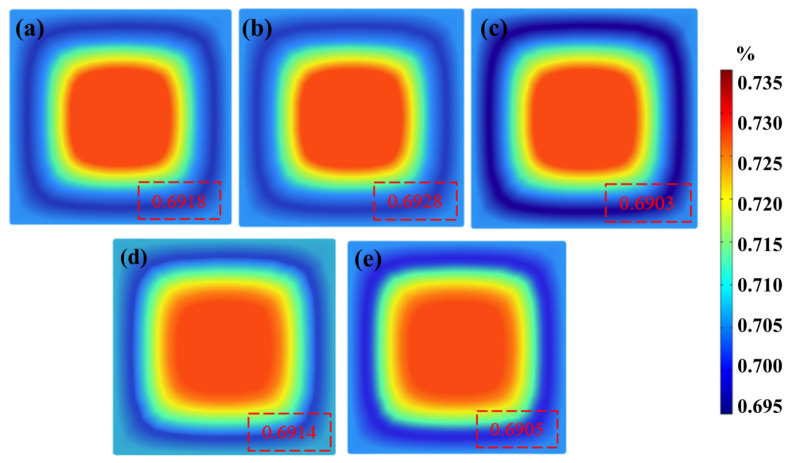
Solute distribution at mold outlet section under different M−EMS parameters: (**a**) 3 Hz + 100 A; (**b**) 3 Hz + 300 A; (**c**) 3 Hz + 500 A; (**d**) 4 Hz + 300 A; (**e**) 5 Hz + 300 A.

Figure 8 shows the solute distribution cloud of the mold outlet section under different M−EMS parameters, where blue represents low solute concentration, and red represents high solute concentration. As the M−EMS current is continuously increased from 100 A to 300 A, the subcutaneous negative segregation of carbon is improved, and the solute mass percentage increases from 0.6918% to 0.6928%. With the continuous increase in M−EMS current from 300 A to 500 A, the negative segregation degree of the billet increases accordingly, and the solute mass percentage decreases from 0.6928% to 0.6903%. This is because the molten steel’s temperature drops rapidly near the mold walls, and the solute transfer decreases. Meanwhile, the rotational velocity continues to increase with the continuous accumulation of electromagnetic force on the molten steel. As a result, the solute at the interface between the high− and the low−temperature regions is unable to transfer to the low−concentration region, forming a negative segregation zone under the skin of the billet.

Compared with Figure 8b,d,e, the solute carbon concentration in the subcutaneous area of the casting billet decreases from 0.6928% to 0.6905% with the increase in frequency from 3 Hz to 5 Hz. This may be caused by the electromagnetic stirrer’s force on the molten steel being limited by the depth of penetration of the electromagnetic field. In the case of a fixed electromagnetic stirring current, the depth of electromagnetic force on molten steel decreases with increasing frequency. Therefore, the effect of electromagnetic forces on the molten steel is minor at a higher stirring frequencies. At a frequency of 3 Hz, the electromagnetic force significantly affects the horizontal rotating flow driven by the electromagnetic force. Then, M−EMS can effectively stir the molten steel and thoroughly mix the flow to reduce the negative segregation.

**Figure 9:**
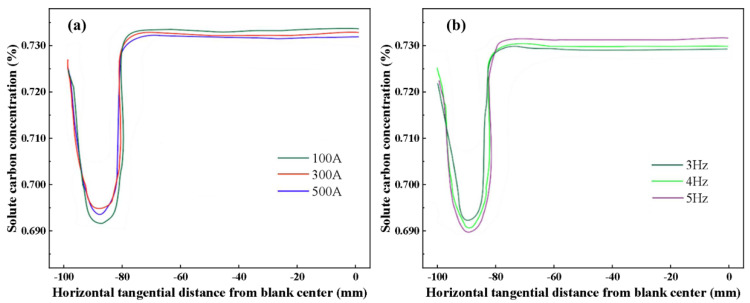
Solute carbon distribution at the horizontal center of mold outlet: (**a**) Current at 100, 300, 500 A of 3 Hz; (**b**) frequency at 3, 4, 5 Hz of 300 A.

Figure 9 shows solute carbon distribution in the horizontal center of the mold outlet under different M−EMS parameters. As the steel enters the inner cavity of the mold from the entrance, the solute diffuses from the center to the surrounding walls, and the center of the mold always maintains a high carbon concentration due to the continuous inflow of molten steel. As the distance from the center of the slab decreases, the solute carbon concentration initially decreases significantly and then increases rapidly. Negative segregation degree is largest for a current of 500 A, followed by a current of 100 A. The negative segregation degree is the lowest with a current of 300 A. When the electromagnetic stirring frequency is specific, the stirring power is small at a current of 100 A, and the molten steel can not be thoroughly stirred. When the current is 500 A, the power is overly large, and the electromagnetic force causes an over−stirring of the molten steel. The same trend variation was also reported in Tao et al.’s experimental study [10]. Since the horizontal rotation of the molten steel driven by M−EMS is significant, the scouring effect on the solid–liquid phase interface of the casting billet is increased. As a result, the solute is carried away from the solid–liquid interface, leading to negative segregation below the casting billet. The effect of the electromagnetic force driving the horizontal rotation of the molten steel is too significant, which will increase the scouring effect on the solid–liquid phase interface of the casting billet [33,34]. Therefore, the solute between the solid–liquid interface is taken away, resulting in negative segregation under the casting billet. In addition, the washed carbon accumulates inside the billet as the solidification process progresses, resulting in the high carbon level in the center of the billet shown in Figure 9. Based on the numerical simulations of the carbon distribution shown in Figure 8 and Figure 9, the appropriate M−EMS parameters are a current of 300 A and a frequency of 3 Hz, which can improve the subcutaneous carbon negative segregation of the casting billet.

### 4.5. Results for Solute Carbon inside Billet

**Figure 10:**
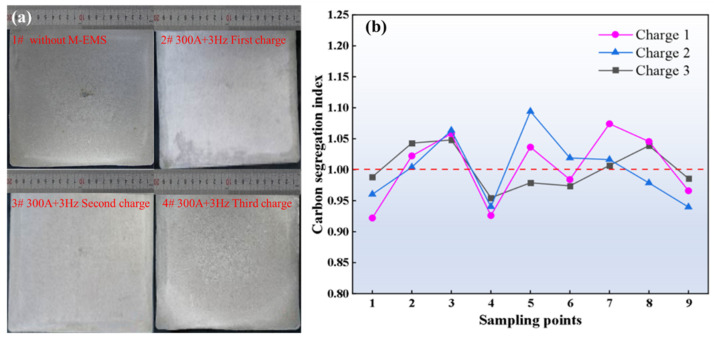
(**a**) Low magnification photograph of the billets obtained at a speed of 1.3 m/min and superheat of 30 °C: 1#—without M−EMS, 2#/3#/4#—three charge with M−EMS at 3 Hz + 300 A; (**b**) analysis results of carbon segregation index of the cross−section of the casting billet.

The conclusions of the previous numerical simulation studies are an essential basis for optimizing casting practice, and it is necessary to optimize continuous casting production using numerically optimal parameters and to verify the effect of reducing carbon sequestration. Due to the focus of the present study on the carbon segregation variation with the action of electromagnetic stirring in the mold, four furnace casting billet samples with or without M−EMS were used for comparative study under a casting speed of 1.3 m/min and superheat degree of 30 °C. According to the optimal parameters of the numerical simulation, the billet cross−sections with 10 mm thickness were collected under steady casting conditions with or without M−EMS. The low−magnification photographs of the collected billets are shown in Figure 10a. A slight negative segregation (bright white) band can be seen in the billet without M−EMS, as shown in Figure 10a (1#). Then, the negative segregation band disappears in a section of the billet obtained under the M−EMS parameter of 3 Hz + 300 A, as shown in Figure 10a (2#,3#,4#). As seen in Figure 10a of the carbon segregation index analysis, the billet shows negative segregation at the edges, positive segregation at 1/2 radius, negative segregation from 1/2 radius to the center, and positive segregation at the center. In order to evaluate the effect of the M−EMS parameters recommended by the numerical model, the carbon segregation index was calculated using Equation (12), with the sampling scheme as shown in Figure 2. According to the carbon concentration of different positions of the billet section shown in Figure 10b, the segregation index of the billet section is calculated to be 0.92~1.10 at the M−EMS parameter of 3 Hz + 300 A. Since the formation of carbon segregation in continuous casting is a highly complex process, and this study focuses only on the effects of M−EMS, there are still some deviations between the results of numerical simulations and the production practice. The extreme difference between the maximum value of the segregation index minus the minimum value is 0.030%, which implies that the segregation of the billet is better optimized under the combination of the mixing parameters. This indicates that 300 A + 3 Hz is an appropriate M−EMS parameter to ameliorate carbon segregation in a billet with a size of 200 mm × 200 mm.

## 5. Conclusions

(1) With the increase in M−EMS frequency from 3 Hz to 5 Hz, the high−temperature zone is shifted upward, which is conducive to the rapid solidification of the cast billet. At the same time, as the M−EMS current increases from 100 A to 500 A, the maximum horizontal tangential velocity of the molten steel increases from 0.19 m/s to 0.38 m/s, which promotes the rotational flow of the steel in the horizontal plane.
(2) When M−EMS is applied, the temperature of the molten steel near the mold walls decreases and the flow velocity near the mold walls increases. Hence, the wash−out effect on the initial solid created by the fluid flow and the variation of the solute transport rate produces a trend in the concentration of solute carbon first of a significant decrease and then of a rapid increase, with decreases in distance from the billet center.
(3) When the M−EMS current is 300 A at a frequency of 3 Hz, producing a suitable mixing intensity and suitable current penetration depth effect, the negative segregation control of the carbon solute is controlled optimally. The carbon segregation index of the billet section is controlled at 0.92~1.1.

## Figures and Tables

**Table 1:** Continuous casting parameters and physical properties of molten steel [22,25,26].

Parameter	Value	Parameter	Value
Nozzle inner diameter/mm	40	Equilibrium partition coefficient	0.34
Nozzle outer diameter/mm	60	Magnetic permeability/H·s^−1^	1.257 × 10^−6^
Depth/mm	120	Electric conductivity/S·m^−1^	7.14 × 10^5^
Length of mold/mm	900	Latent heat of fusion/J·kg^−1^	271,000
Casting speed/m·min^−1^	1.3	Solidus temperature/K	1762
Frequency/Hz	3, 4, 5	Liquidus temperature/K	1783
Current/A	100, 300, 500	Diffusion coefficient of carbon in liquid phase/cm^2^·s^−1^	0.0052exp($\frac{-11700}{8.314\cdot T}$)
Inlet temperature/K	1858	Surface emissivity	0.8
Density of molten steel/kg/m^3^	−0.823 × T + 7100	Constant pressure heat capacity of molten steel/J·(kg·K)^−1^	0.0071 × T^2^ − 15.255 × T + 8959
Dynamic viscosity of molten steel/Pa·s	0.0065	Thermal conductivity of molten steel/W·(m·K)^−1^	10^−5^ × T^2^ − 0.033 × T + 50.265

**Table 2:** The main chemical components of 30Cr13 steel (wt.%).

C	Mn	P	S	Si	Cr	Ni
0.725	0.3~0.5	≤0.035	≤0.008	0.25–0.50	12.10–12.60	≤0.3

## Data Availability

The data presented in this study are available on request from the corresponding author.
